# Examining air pollution (PM_10_), mental health and well-being in a representative German sample

**DOI:** 10.1038/s41598-021-93773-w

**Published:** 2021-09-16

**Authors:** Katja Petrowski, Stefan Bührer, Bernhard Strauß, Oliver Decker, Elmar Brähler

**Affiliations:** 1 Medical Psychology and Medical Sociology, University Medical Center of the Johannes Gutenberg University of Mainz, Duesbergweg 6, Mainz, Germany; 2 Department of Psychosomatic Medicine and Psychotherapy, University Medical Center of the Johannes Gutenberg University of Mainz, Untere Zahlbacher Str. 8, 55131 Mainz, Germany; 3 Institute of Psychosocial Medicine and Psychotherapy, University Hospital Jena, Jena, Germany; 4 Department of Medical Psychology and Medical Sociology, University of Leipzig, Leipzig, Germany; 5 Integrated Research and Treatment Center (IFB) Adiposity Diseases-Behavioral Medicine, Psychosomatic Medicine and Psychotherapy, University of Leipzig Medical Center, Leipzig, Germany

**Keywords:** Psychology, Environmental sciences, Health care, Risk factors

## Abstract

There is a growing debate on the role of the physical environment and what constitute risk and protective factors for mental health. Various forms of air pollution have shown links to physical and mental health concerns and considering that Germany does not meet the WHO air quality standards—poor air quality affects a large proportion of Germans and is more important now than ever. This study investigates the physical environmental factor, air pollution, measured by particulate matter of particles with an aerodynamic diameter smaller than 10 µm (PM_10_) and effects on determinants of mental health and well-being (life satisfaction, stress resilience, anxiety, depression, and self-esteem). A representative sample of N = 3020 German adults with 54% females (46% males) and an age range between 18 and 92 years (M = 49.04, S.D. ± 17.27) was used. Multivariate linear regression analyses show that higher life satisfaction, more self-esteem and higher stress resilience are predicted by less air pollution (PM_10_). Individual income, age, and gender were taken into account for each regression model. Gender specific sub-analyses revealed similar predictions for PM_10_ and stress resilience whereas PM_10_ and self-esteem were only significantly associated for females. Associations between mental health or well-being determinants and air pollution (PM_10_) are found in the representative German sample.

## Introduction

In a recent study of global diseases, injuries and risk factors, ambient air pollution is one of three leading environmental risk factors since the 1990s and has remained important among the leading ten risk factors for men and women^1,2^. Thus, global air pollution is a major concern in societies and evokes environmental movements. Many would agree it is beneficial to foster more sustainable solutions to protect nature and stabilize the climate (e.g. environmentally friendly mobility solutions, farming, energy supply etc.), especially as existing harmful air pollution affects public health^1–4^. Hence air pollution is considered a major risk factor especially for cardiovascular and respiratory morbidity and mortality^1,4^. Vehicles burning fossil fuels, waterway transportation, biomass combustion, aircrafts, agriculture, and industry power plants^2,3^ are the main contributors to atmospheric pollution. Possible health impacts of such air pollution sources are often related to particulate matter (PM), as air quality levels (including PM_2.5–10_, nitrogen dioxide NO_2_, Ozone O_3_, sulfur dioxide SO_2_ and carbon monoxide CO) can be measured worldwide, data are often publicly available and have been consistently linked to adverse health effects^4–6^. In 2010, ambient PM pollution was ranked ninth among the leading risk factors for global disease burden and accounted for 3.1 million deaths worldwide due to respiratory, cardiovascular and cerebrovascular diseases^5^. In 2016, this figure rose globally to approximately 4 million deaths attributable to air pollution^3^. Thus, it remains highly relevant to improve knowledge on air pollution as a risk factor to public health and to explore associated psychological factors. This would help to better understand biopsychosocial impacts of air pollution to reduce adverse health effects.

Epidemiological studies have addressed the associations between air pollution and premature mortality or reduced life expectancy due to cardiovascular and respiratory diseases (e.g. lung cancer, chronic bronchitis, or asthmatic attacks)^7–12^. Additional indirect effects of air pollution may occur including irritation, annoyance, displeasure, and less physical outdoor activities^13^. While studies have addressed psychological well-being indexes of air pollution in humans^14–20^, few benefit from large sample sizes or nationally representative data^21–23^. It has even been hypothesized that air pollution may increase the risk of psychiatric disorders such as schizophrenia and other psychotic disorders^24^, especially during the initial years of an individual’s life^19^. Hereby, an increase in the number or air particles is associated with an increase in psychiatric emergency units and mental distress^20,25^. Daily and even long-term exposure to air pollution is a risk factor for generic mental health disorders ^26–29^ such as depressive disorders, suicidal ideation and substance abuse^30–32^. Besides the development of disease and mental disorders, a German representative study in 2017 found air pollution (PM_2.5_) as a significant predictor of chronic stress in healthy individuals (higher PM_2.5_ values—more chronic stress symptoms) after controlling for individual income and age^21^. This is concerning considering that air pollution in most German urban areas is still above the standard values recommended by the World Health Organization^8^. According to the German Federal Environmental Agency (Umweltbundesamt-UBA), PM_10_ emission with particles smaller than 10 µm have reduced from 0.33 million tons in 1995 to 0.2 million tons in 2017^33^. Even though there is this decrease in PM_10_ in Germany, lower concentrations of PM_10_ exposure still show negative effects on public health^7^. Air pollution is the largest environmental risk factor for human health^1^, however, mental health findings specifically on depression and anxiety are inconsistent^22,23,35–36^.

Recently, there has been a growing body of literature providing evidence for the positive link between physical or built environment and mental health^22,23,35,37,38^. Mental health generally refers to a state of emotional and psychological well-being to cope with daily demands^39^. The built environment, in terms of green infrastructure, has been found to be a protective factor to psychological well-being^23,40,41^. In contrast, outdoor pollutants have the reverse effect as people engage less in physical outdoor activities^13,23^ or the positive effect of physical activity is inhibited^42^. Additional findings showed that increased pollution (PM_2.5_) contributes to the probability of being chronically stressed^21^ or PM_10_ being associated with higher COVID-19 mortality^43^, however more research is required to have robust evidence. More importantly the negative effects of air pollution influence individuals differently. Vulnerable populations (e.g. infants, elderly, or individuals with pre-existing conditions)^3^ as well as individuals who struggle with coping may be disproportionally affected. This is referred to as the adaptive cost hypothesis^44^ where resources to cope with environmental demands may interfere with other adaptive processes (e.g. psychosocial stressors). Therefore, this study investigates links between air pollution (PM_10_) and subjective mental health and well-being determinants (depression, anxiety, stress resilience, life satisfaction, self-esteem) in a representative German sample. Since existing literature has mainly focused on effects of air pollution on mental health outcomes of depression and anxiety^30,45–47^, this link is examined in this study to provide more empirical grounding. Additionally, air pollution and its effect on an individual’s stress resilience is included since literature describes its links theoretically but empirical findings are very rare. Stress resilience refers to the adaption to ongoing life challenges in order to quickly recover and promote mental health^48^. Thus, it appears that one’s stress resilience or the opposite, one’s vulnerability, may be associated with environmental stressors such as daily air pollution exposure. In this context of environmental stressors, it is not clear which mechanisms improve mental health. It is well-known that air pollution can activate the hypothalamic–pituitary–adrenal (HPA) axis and overlapping conditions between stress axis regulation and adverse health effects of air pollutants exist^4,49^. Continuous exposure to air pollution or exposure during vulnerable life phases may lead to allostatic load and/or disfunction of the HPA axis and, as conceptualized by McEwen^50–52^, ultimately affect physiological stress response systems^4^. Thus, a reduced air pollution at individual, local, or regional levels may decrease vulnerability to adverse health impacts, but requires more consistent findings^4^. The magnitude of air pollution exposure can and should be addressed by policy makers.

## Methods

### Sample

The data was gathered by the USUMA (Independent service for surveys, methods, and analyses) and the Berlin Polling Institute in 2006, who selected households and participants by random-route sampling^53^. The total sample size of adults was N = 3020. All participants volunteered and received a data protection declaration in agreement with the Helsinki Declaration. The study was conducted in accordance with the ethics guidelines of the German Professional Institutions for Social Research. The representative data was approved by the Ethical Committee of the Medical Faculty, University of Leipzig, Germany, in accordance with ethical, medical-scientific, and legal guidelines (418-17-EK). Written informed consent was obtained by all participants.

The air pollution (PM_10_) data was directly retrieved from the German Environment Agency^33^. The UBA measured daily to create the 2006 yearly average of pollutant exposure in microgram per cubic meter (µg/m^3^). Smaller fine dust particles of particulate matter 2.5 µg/m^3^ (PM_2.5_) were not collected nationwide across regions until 2008 and was therefore not included. A total of 428 measurements stations collected daily pollution data. The representative dataset was merged with the 2006 environmental data based on official German district codes (GKZ-amtliche Gemeindekennziffer). The GKZ numbers of two states (Bundesländer) Saxony and Saxony-Anhalt have changed, which required manually matching participants with the correct GKZ. Across disciplines there are many dimensions to measure mental health and well-being^54^. In the present study, we operationalized determinants of mental health and mental well-being, including stress resilience^55–57^, depression and anxiety symptoms^54,58,59^, self-esteem^60^, and life satisfaction^54,61^, while controlling for gender, age and income^60^.

### Instruments

The shortened German version of the resilience scale was implemented ^56,62^. The original version of the RS is comprised of 25 items. The 11 items of the RS-11 are rated on a seven-point-Likert scale ranging from 1 = “I do not agree” to 7 = “I agree”. Higher total scores on the scale represent high resilience in contrast to low scale values. Internal consistency (Cronbach’s Alpha) for the original samples was reported with α = 0.91 ^56^ indicating very good reliability.

As the assessment of the severity of depressive symptoms, the Patient Health Questionnaire-2 (PHQ-2) was applied ^63^. This ultra-short screening instrument was validated and demonstrated good psychometric properties covering the two main symptoms of major depression a) depressed mood and b) loss of interest, referring to the last two weeks. Response options range from 0 = “not at all” to 3 = “nearly every day”. The PHQ-2 total sum varies from 0 to 6 whereas values ≥ 3 indicate cut-off points between normal range and likely cases of depression.

The General Anxiety Disorder-2 (GAD-2) was implemented to examine the intensity of anxiety symptoms ^64^. The participants were questioned how often they had been bothered by each of the two main symptoms of a generalized anxiety disorder during the previous two weeks that are (a) “nervousness, anxiety, or strain” and (b) “not being able to stop or to control worries”. Response options range from 0 = “not at all” to 3 = “nearly every day”. Similar to PHQ-2, the total sum of the GAD-2 varies from 0 to 6 whereas values ≥ 3 indicate cut-off points between normal range and likely cases of anxiety.

The German adapted version^60^ of the Rosenberg’s Self-Esteem scale (RSES) from Ferring and Filipp^65^ was administered. The RSES consists of five positively and five negatively worded items rated on a 6-point Likert scale. Subjects indicate to what extent the items describe them and the scale ranges from “1” = strongly disagree to “6” = strongly agree. Negatively worded items 2, 5, 6, 8, and 9 were reversed and summed up to the negative self-image subscale. The sum of the remaining items represents the positive self-image. Both subscales were summed up to create the global self-esteem score.

The German version of the General Life Satisfaction questionnaire (FLZM—Allgemeine Lebenszufriedenheit) was used^61,66^. The general module of the FLZM consists of eight items from various areas of life that are subjectively rated on a five-point Likert scale. The areas include friends, hobbies/leisure time, health, income/financial security, occupation/work, living situation, family/children, relationship/sexuality that are individually weighted by the subjects. The rating and weighting ranges from (1 = not important/unsatisfied, to 5 = extremely important/very satisfied). The combination of both required the recoding of importance of each item and the satisfaction calculation using this formula (importance − 1) × (satisfaction × 2 − 5). Each coded item for importance and satisfaction was multiplied; these products were summed up for the global weighted life satisfaction score. This global score ranges from − 96 to + 160. Higher values indicate a higher weighted life satisfaction.

### Statistical procedure

Data analysis was performed using SPSS statistics 23 version 5. Sample characteristics were analyzed using mean values, standard deviations, and frequencies. Multivariate linear regressions were calculated to predict the influence of air pollution PM_10_ on subjective mental well-being determinants (RS-11, PHQ-2, GAD-2, RSES, FLZM). For the regression analyses the goodness of fit, regression coefficients, the 95% confidence intervals for the unstandardized regression coefficients, significant levels and t-statistics were calculated. Further included factors were age and individual income^67–69^. We calculated models for the entire sample controlling for age and income. Independent analyses on gender sub-samples are conducted to better understand gender effects.

## Results

This study included N = 3020 adults living in Germany. Females represent 54% (46% males) of the sample. Women were slightly overrepresented with 54.04 versus 51.04%^70^ in the German population. Participants range in age from 18 to 92 years (M = 49.04, S.D. ± 17.27). A socio demographic overview and list of used variables is provided in Table 1.

**Table 1:** Study variables and descriptive statistics.

Variables	Valid cases	Minimum	Maximum	Mean	SD
**Demographic factors**					
Gender	3020			1.54	0.49
Male	1389			46.00%^P^	
Female	1631			54.00%^P^	
Age	3020	18	92	49.04	17.27
**Individual income**	2618 (86.8%)^P^			7.09	3.21
< 750€	535			(17.07%)^P^	
750€ bis < 1250€	943			(31.2%)^P^	
1250€ bis < 2000€	834			(27.6%)^P^	
2000€ bis < 3500€	271			(9.0%)^P^	
≥ 3500	35			(1.2%)^P^	
Not responded	402			(13.2%)^P^	
**Pollution**					
PM_10_: average annual large particulate matter exposure μg/m^3^	3020	13	39	26.83	5.05
**Mental well-being**					
Resilience (RS-11)	3020	14	77	59.30	10.60
Life satisfaction	2934	− 78	160	60.57	36.09
Anxiety (GAD-2)	3016	0	6	0. 85	1.13
Depression (PHQ-2)	3010	0	6	0.97	1.23
Self-Esteem (RSES)	3018	2.00	60.00	48.72	8.60

*SD* standard deviation, *P* percentage of total sample or sub sample, *RS* resilience scale 11-items, *GAD* General Anxiety Disorder 2-items, *PHQ-2* Patient Health Questionnaire 2-items, *RSES* Rosenbergs’s Self-Esteem Scale.

Multivariate linear regression models were applied in order to compare how each model predicts the influence of air pollution PM_10_ on anxiety, depression, stress resilience, life satisfaction and self-esteem as mental health determinants.

First, the full sample was examined for each mental health determinant. Previous effects of air pollution on depression or anxiety are inconsistent; the current results showed that PM_10_ was not significantly associated with anxiety (p = 0.181) nor depression (p = 0.283). Models with PM_10_ on stress resilience (p < 0.001), life satisfaction (p = 0.012) and self-esteem (p = 0.011) showed significant results without gender specific analyses. Age and income were significant predictors in all models except age for self-esteem (p = 0.177). The model predicting stress resilience explained the highest amount of variance with 8.2%, followed by the model of life satisfaction with 5.7% explained variation. A microgram per cubic meter (µg/m^3^) increase in annual air pollution (PM_10_) exposure is associated with a 0.34 decrease in one’s subjective life satisfaction score ceteris paribus (see Table 2).

**Table 2﻿:** Regression analyses predicting mental health and well-being determinants.

Model/variables	Unstandardized coefficient (SE)	Standardized regression coefficient	t-statistics	p-value	Adjusted R-squared
**Anxiety**					0.019
Age	0.003 (0.00)	0.045	2.33	0.019*	
Income	− 0.161 (0.02)	− 0.136	− 7.03	< 0.001***	
PM_10_	0.006 (0.00)	0.026	1.33	0.181	
**Depression**					0.027
Age	0.005 (0.00)	0.06	3.53	< 0.001***	
Income	0.202 (0.02)	− 0.15	− 8.13	< 0.001*	
PM_10_	0.005 (0.00)	0.02	1.07	0.283	
**Stress resilience**					0.082
Age	− 0.13 (0.01)	− 0.21	− 11.42	< 0.001***	
Income	2.13 (0.20)	0.19	10.33	< 0.001***	
PM_10_	− 0.15 (0.03)	− 0.07	− 3.88	< 0.001***	
**Life satisfaction**					0.057
Age	− 0.27 (0.04)	− 0.13	− 6.85	< 0.001***	
Income	7.60 (0.72)	0.20	10.52	< 0.001***	
PM_10_	− 0.34 (0.13)	− 0.04	− 2.50	0.012*	
**Self-esteem**					0.023
Age	− 0.01 (0.01)	− 0.02	− 1.35	0.177	
Income	1.32 (0.17)	0.14	7.68	< 0.001***	
PM_10_	− 0.08 (0.03)	− 0.04	− 2.54	0.011*	

Dependent variables anxiety, depression, stress resilience, life satisfaction and self-esteem.

*PM*_*10*_ particulate matter 10 µg/m^3^, *SE* standard error.

*p < 0.05, ** p < 0.01, *** p < 0.001.

Secondly, gender specific regression models were conducted and are presented in Table 3 for males and Table 4 for females. For males, age and PM_10_ were not significant in predicting anxiety, depression, or self-esteem and only income was significant. Age (p < 0.001), income (p < 0.001) and PM_10_ (p = 0.004) are significant predictors of stress resilience explaining 8.5% of the variance. For life satisfaction, only age (p = 0.005) and income (p < 0.001) were found to be significant.

**Table 3:** Regression analyses predicting mental health and well-being determinants for males.

Model/variables	Unstandardized coefficient (SE)	Standardized regression coefficient	t-statistics	p-value	Adjusted R-squared
**Anxiety**					0.009
Age	0.00 (0.00)	0.03	1.30	0.19	
Income	− 0.11 (0.03)	− 0.10	− 3.68	< 0.001***	
PM_10_	− 0.00 (0.00)	− 0.00	− 0.13	0.893	
**Depression**					0.028
Age	0.00 (0.00)	0.01	0.44	0.658	
Income	− 0.21 (0.03)	− 0.17	− 6.34	< 0.001***	
PM_10_	0.00 (0.00)	0.00	0.33	0.737	
**Stress resilience**					0.085
Age	− 0.01 (0.01)	− 0.18	− 6.72	< 0.001***	
Income	2.50 (0.28)	0.23	8.70	< 0.001***	
PM_10_	− 0.15 (0.05)	− 0.07	− 2.87	0.004**	
**Life satisfaction**					0.124
Age	− 0.15 (0.05)	− 0.07	− 2.81	0.005**	
Income	12.94 (0.98)	0.34	13.17	< 0.001***	
PM_10_	− 0.24 (0.18)	− 0.03	− 1.30	0.193	
**Self-esteem**					0.050
Age	− 0.00 (0.01)	− 0.00	− 0.22	0.826	
Income	2.01 (0.24)	0.22	8.34	< 0.001***	
PM_10_	− 0.04 (0.04)	− 0.02	− 1.03	0.299	

Dependent variables anxiety, depression, stress resilience, life satisfaction and self-esteem.

*PM*_*10*_ particulate matter 10 µg/m^3^, *SE* standard error.

*p < 0.05, ** p < 0.01, *** p < 0.001.

**Table 4:** Regression analyses predicting mental health and well-being determinants for females.

Model/variables	Unstandardized coefficient (SE)	Standardized regression coefficient	t-statistics	p-value	Adjusted R-squared
**Anxiety**					0.013
Age	0.00 (0.00)	0.05	1.80	0.067	
Income	− 0.15 (0.03)	− 0.10	− 4.00	< 0.001***	
PM_10_	− 0.01 (0.00)	− 0.05	1.82	0.069	
**Depression**					0.022
Age	0.008 (0.00)	0.11	4.22	< 0.001***	
Income	− 0.16 (0.04)	− 0.10	− 3.95	< 0.001***	
PM_10_	0.00 (0.00)	0.02	1.00	0.315	
**Stress resilience**					0.081
Age	− 0.15 (0.01)	− 0.24	− 9.35	< 0.001***	
Income	1.96 (0.33)	0.15	5.78	< 0.001***	
PM_10_	− 0.13 (0.05)	− 0.06	− 2.54	0.011*	
**Life satisfaction**					0.037
Age	− 0.39 (0.05)	− 0.18	− 6.79	< 0.001***	
Income	2.75 (1.18)	0.06	2.32	0.02*	
PM_10_	− 0.34 (0.19)	− 0.04	− 1.80	0.07	
**Self-esteem**					0.009
Age	− 0.02 (0.01)	− 0.04	− 1.67	0.095	
Income	0.80 (0.28)	0.07	2.86	0.004**	
PM_10_	− 0.10 (0.04)	− 0.06	− 2.29	0.022*	

Dependent variables anxiety, depression, stress resilience, life satisfaction and self-esteem.

*PM*_*10*_ particulate matter 10 µg/m^3^, *SE* standard error.

*p < 0.05, ** p < 0.01, *** p < 0.001.

Results for females differ slightly compared to those of males. Similar to males, income alone significantly predicted anxiety (p < 0.001). In the depression model for females, age and income were found to be highly significant (p < 0.001). Comparable to males, the stress resilience model of females is predicted by age (p < 0.001), income (p < 0.001), and PM_10_ (p = 0.011), of which, explains 8.1% of variance. As for males, life satisfaction is found to be predicted by age (p < 0.001) and income (p = 0.02). In contrast to the male model, self-esteem for females was significantly predicted by income (p = 0.004). and PM_10_ (p < 0.022), however, the adjusted R^2^ indicates a low proportion of explained variance.

A microgram per cubic meter (µg/m^3^) increase in annual air pollution (PM_10_) exposure is associated with a 0.15 (males) or 0.13 (females) decrease in one’s subjective stress resilience score ceteris paribus (see Tables 3 and 4).

## Discussion

This study uses nationally representative data of the German population along with air pollution (PM_10_) to analyze the link to mental health and well-being. Although previous studies have shown connections between mental health or well-being and air pollution^5,23,35,36^, there are also some inconsistent effects^21–23,35,37,38,40,41^.

Results do not show strong associations between air pollution and mental health determinants^23,35,36^. Previous literature is inconsistent in terms of associations between air pollution and depression or anxiety. Our results on depression symptoms and PM_10_ is in line with some earlier findings showing no significant associations^22,34^ and rather contradict other studies^5,23,35,36^. For instance, in a sample of four countries including Germany, no consistent evidence was found for associations between air pollution (PM_2.5_ and PM_10_) and depressed mood^22^, while others found a significant link between air pollution (PM_10_ or PM_2.5_) and depressive symptoms in Asia^5^ and the United States^35^. Comparable to previous findings, we did not find associations between air pollution and anxiety symptoms. This may be in part due to the type of air pollution measured, as anxiety symptoms were mainly reported for PM_2.5_^35,36^, but not for larger particles^36^.

Evans et al.^44^ mentioned that exposure to air pollution reduces the coping ability against stress as it might affect fatigue, helplessness, and anxiety, which ultimately increases an individual's vulnerability^15^. This is confirmed by an animal study showing that chronic stress may increase susceptibility to impacts of air pollution^71^. This proposed effect of vulnerability is shown by the present results on subjective stress resilience. After controlling for income and age, pollution shows a significant predictive value on stress resilience. In the present study, those with the highest subjective stress resilience are young individuals with higher income and lower air pollution (PM_10_) exposure. This is in line with previous results showing associations between fine dust (PM_2.5_) particles on chronic stress^21^, whereby higher fine dust exposure was associated with higher reported chronic stress by the participants. In the long run this might lead to lower stress resilience or an inhibited stress coping ability.

The present results show a small effect for life satisfaction. Living in less polluted areas is associated with higher individual life satisfaction. This effect is visible even after controlling for income and age. No gender specific models are found to be significant for life satisfaction, anxiety, or depression.

A previous study has shown that individuals with higher self-esteem rarely use avoidance coping strategies^55^. Therefore, men and women in the current study may apply different coping strategies against environmental stressors, which provides one possible explanation for the gender differences in self-esteem. Unfortunately, specific coping strategies were not examined in the current study. Research on coping strategies might be promising to promote stress resilience and counteract different environmental stressors such as air pollution. In particular, coping with environmental demands and whether such an adaption interferes with other adaptive processes (e.g. physical stress response systems) is of interest. Self-esteem as well as life satisfaction have shown intercorrelations to stress resilience^57^ and our study results show differences in terms of age and PM_10_ as predictors.

In interpreting the results with caution, it is important to recognize the limitations of this study. Since both data sets are cross-sectional and thus represent one moment in time, further analyses on long term effects cannot be drawn. Our study used self-reported measurement scales, but objective measures or confirmed assessments by health professionals can prevent potential misclassification. Data was collected for a different focus and that limits the types of insights and analyses. Measurement stations of particulate matter (PM_10_ and PM_2.5_) are still regionally limited across Germany. Hence it is likely that a participant’s PM_10_ exposure varies from others even when living in the same regional districts. Based on geographical regions and limited measurement stations, it is difficult to entirely capture the pollution exposure of one participant on a smaller or even individual level. This is a common problem and bias of misclassification mentioned in many air pollution studies^22,23,34–36^. In order to allocate pollution exposure values to participants at the smallest possible level, advanced methods are required. For instance, long-term and accurate exposure data could be collected by applying individual mobility tracing via smartphones. However, mobility tracing, additional weather aspects and individual pollution measures are very costly, but would improve robustness of evidence.

A major strength of the present study is the large representative data collection, including subjective psychological parameters, which was matched with reliable pollution data (PM_10_). Future research with newer data should include PM_10_ and other available air pollution indicators (e.g. PM_2.5_, NO_2_, O_3_) along with noise, physical activity, and other indirect effects. Although combined exposure models exist, more robust results using longitudinal designs would be beneficial. The COVID-19 pandemic shows that longitudinal studies are necessary to examine long-term effects on physical health^43^ (e.g. long COVID symptoms) and mental health. On the one hand, elevated levels of air pollution, particularly in urban areas, show respiratory impairments that could increase the risk of more severe COVID-19 disease progression^43^, and on the other hand, air pollution may have a compounding effect together with viral diseases on mental health. For instance, climate change, air pollution, and the COVID-19 pandemic are expected to have effects on mental health, specifically on anxiety and depression, stress/trauma-related disorders, and substance abuse^72^. Additionally, positive effects of COVID-19 and air pollution (e.g. lower emissions during lockdown) on mental health should be considered. This may guide future research to investigate joint effects of air pollution, urbanization, and infectious viral diseases on mental health^43,72^. Future longitudinal studies on mental health, specifically on stress resilience processes, require measures of pre and post states of general health, major negative life events and daily hassles including accurate air pollution exposure times. Translations from animal studies are important, but laboratory experiments on humans with polluted air might be challenging and require ethical consideration. In an animal study, chronic social stress and susceptibility to concentrated ambient fine particles in rats were examined^73^. Effects of chronic stress and air pollution elevated biomarkers such as breathing frequency, shorter inhalation and exhalation times, increased inflammatory biomarkers of the C-reactive protein and the numbers of lymphocytes and monocytes^73^. In terms of translational research, biomarkers should be further examined in humans to explore whether findings can be replicated.

In addition to findings of previous research on chronic stress and stress resilience, other factors such as work stress should be specified when looking at air pollution. Our study shows some associations between common mental health factors (e.g. stress resilience, life satisfaction, self-esteem) and air pollution (PM_10_) in a representative German sample. Due to the fact that Germany is not reaching the WHO air quality standards yet, it is important to highlight that poor air quality affects a large amount of Germans. Although effects of PM_10_ on individual mental health determinants in the current study are small, the ramifications on the general German population and health system can be noteworthy. Furthermore, inflammation and oxidative stress are considered as central pathophysiological mechanisms by which air pollution induces brain damage^2^. Therefore, politicians and policy makers, psychologists, and experts on occupational and environmental medicine should account for found effects of air pollution on mental health and vulnerability.

## Data Availability

The representative data set is not publicly available. Air pollution data can be retrieved from: https://www.umweltbundesamt.de/daten/luft/luftdaten/jahresbilanzen/eJxrWpScv9BwUWXqEiMDAzMAMK8FtQ==.
